# Pérdida en el seguimiento de pacientes tratados por tuberculosis resistente a rifampicina o multidrogorresistente en Ecuador

**DOI:** 10.26633/RPSP.2019.91

**Published:** 2019-12-20

**Authors:** Nelly Tatés-Ortega, Jorge Álvarez, Lucelly López, Alberto Mendoza-Ticona, Edith Alarcón-Arrascue

**Affiliations:** 1 Estrategia Zonal de Prevención y Control de Tuberculosis Coordinación Zonal 9-Salud, Ministerio de Salud Pública del Ecuador Ecuador Estrategia Zonal de Prevención y Control de Tuberculosis. Coordinación Zonal 9-Salud. Ministerio de Salud Pública del Ecuador, Ecuador.; 2 Vigilancia Epidemiológica Zonal Coordinación Zonal 9-Salud, Ministerio de Salud Pública del Ecuador Ecuador Vigilancia Epidemiológica Zonal. Coordinación Zonal 9-Salud. Ministerio de Salud Pública del Ecuador, Ecuador.; 3 Universidad Pontificia Bolivariana Universidad Pontificia Bolivariana Medellín Colombia Universidad Pontificia Bolivariana, Medellín, Colombia.; 4 Universidad Nacional de San Agustín Universidad Nacional de San Agustín Arequipa Perú Universidad Nacional de San Agustín, Arequipa, Perú.; 5 Organización Panamericana de la Salud Organización Panamericana de la Salud Washington D.C. Estados Unidos de América Organización Panamericana de la Salud, Washington D.C., Estados Unidos de América.

**Keywords:** Tuberculosis multidrogorresistente, pérdida en el seguimiento, tratamiento, investigación operativa, Ecuador, Multidrug-resistant tuberculosis, lost to follow-up, therapeutics, operations research, Ecuador, Tuberculose resistente a múltiplos medicamentos, perda de seguimiento, terapêutica, pesquisa operacional, Ecuador

## Abstract

**Objetivo.:**

Determinar la incidencia de pérdida en el seguimiento (PEES) en pacientes tratados por tuberculosis (TB) resistente a rifampicina o multidrogorresistente (TB-RR/MDR) y los factores asociados a esta condición de egreso en Ecuador.

**Métodos.:**

Estudio de cohorte retrospectivo de pacientes con TB-RR/MDR tratados con el esquema de 18 a 24 meses de la Organización Mundial de la Salud en 2014 y 2015 notificados al Ministerio de Salud del Ecuador. Se determinó la incidencia de PEES y se compararon las características clínicas y epidemiológicas de los casos egresados como PEES versus los egresados como éxito de tratamiento. Se analizó la sobrevida con regresión de Cox para evaluar factores asociados a PEES.

**Resultados.:**

De 328 casos, 270 (82,3%) fueron analizados porque tuvieron condición de egreso notificada. El egreso como PEES fue 39,6% y el éxito de tratamiento 50,4%. Los factores de riesgo asociados a PEES fueron: antecedente de egreso como PEES en episodio previo de TB, cociente de riesgos instantáneos (HR, por sus siglas en inglés): 2,96 (1,53-5,73), *P* < 0,001; adicción al alcohol o drogas, HR: 2,82 (1,10-7,23), *P* = 0,031 y tener diagnóstico por la prueba Xpert^®^ (TB-RR), HR: 1,53 (1,0-2,35), *P* = 0,048. Del total de PEES, 43% ocurrió después de nueve meses de tratamiento.

**Conclusión.:**

La incidencia de PEES en pacientes con TB-RR/MDR en Ecuador está por encima del promedio en la Región de las Américas. Los tres factores identificados refuerzan la implementación de regímenes acortados y atención centrada en el paciente, siguiendo la Estrategia Fin a la Tuberculosis.

## Abstract (en)

**Objective.:**

Determine the incidence of loss to follow-up (LTFU) in patients treated for rifampicin-resistant tuberculosis (RR-TB) or multidrug-resistant tuberculosis (RR/MDR-TB), and the factors associated with this discharge status in Ecuador.

**Methods.:**

Retrospective cohort study of patients with RR/MDR-TB who followed the World Health Organization’s 18-24-month treatment regimen in 2014 and 2015, as reported by the Ministry of Health of Ecuador. The incidence of LTFU was determined, and clinical and epidemiological manifestations of cases discharged as LTFU were compared with those discharged as successfully treated. Survival was analyzed with Cox regression in order to evaluate factors associated with LTFU.

**Results.:**

Of 328 cases, 270 (82.3%) were analyzed because they had a reported discharge status. Discharge as LTFU accounted for 39.6% of cases, and as successfully treated, 50.4%. The risk factors associated with LTFU were: previous discharge as LTFU in a previous TB episode [hazard ratio (HR): 2.96 (1.53-5.73), *P* < 0.001]; addiction to alcohol or drugs [HR: 2.82 (1.10-7.23), *P* = 0.031]; and having an Xpert^®^ diagnosis (TB-RR) [HR: 1.53 (1.0-2.35), *P* = 0.048]. Of the total LTFU, 43% occurred after nine months of treatment.

**Conclusion.:**

The incidence of LTFU in patients with RR/MDR-TB in Ecuador is above the average for the Region of the Americas. The three identified factors support implementation of shorter regimens and patient-centered care, in line with the End TB Strategy.

## RESUMO

**Objetivo.:**

Determinar o percentual de perda de seguimento de pacientes tratados para tuberculose resistente à rifampicina (TB-RR) ou tuberculose multirresistente a medicamentos (TB-MR) e os fatores associados à interrupção do tratamento no Equador.

**Métodos.:**

Estudo de coorte retrospectivo de casos de pacientes com TB-RR/TB-MR tratados em 2014 e 2015 com o esquema farmacológico de 18 a 24 meses de duração da Organização Mundial da Saúde (OMS) que foram notificados ao Ministério da Saúde do Equador. Foi determinado o percentual de perda de seguimento e foram comparadas as características clínicas e epidemiológicas dos casos de interrupção do tratamento por perda de seguimento e daqueles com alta por sucesso no tratamento. Uma análise da sobrevida com o modelo de regressão de Cox foi realizada para avaliar os fatores associados à perda de seguimento.

**Resultados.:**

De 328 casos registrados, 270 (82,3%) foram incluídos na análise por terem tido sua interrupção ou alta notificadas. Houve interrupção por perda de seguimento em 39,6% dos casos e alta por sucesso no tratamento em 50,4%. Os fatores de risco associados à perda de seguimento foram: história de perda de seguimento em tratamento anterior de TB, razão de riscos (*hazard ratio*, HR) 2,96 (1,53–5,73, *P* < 0,001); consumo excessivo de álcool ou drogas, HR 2,82 (1,10–7,23, *P* = 0,031); e diagnóstico de tuberculose pelo teste Xpert^®^ (TB-RR), HR 1,53 (1,0–2,35, *P* = 0,048). A perda de seguimento ocorreu após nove meses de tratamento em 43% dos casos.

**Conclusão.:**

O percentual de perda de seguimento de pacientes com TB-RR/TB-MR no Equador está acima da média da Região das Américas. Os três fatores identificados no estudo reforçam ser necessário implementar esquemas de tratamento mais curtos e prestar atenção centrada no paciente, segundo as recomendações da Estratégia pelo Fim da Tuberculose.

La condición de egreso del tratamiento de la tuberculosis (TB) denominada “pérdida en el seguimiento” (PEES) ha reemplazado al término “abandono del tratamiento” con la finalidad de dejar de culpar al paciente de esta decisión y hacer que los servicios de salud asuman la política de tratamiento centrado en el paciente (1). Según la Organización Mundial de la Salud (OMS), la condición de egreso PEES de los casos de TB resistente a rifampicina (TB-RR) y TB multidrogorresistente (TB-MDR, resistente a rifampicina e isoniacida) tratados con el régimen de 18 a 24 meses fue de 14% a nivel mundial en el 2017. En cambio, para la TB sensible, este mismo valor fue de 8% (2). Una proporción alta de egresos como PEES representa un mayor riesgo para la transmisión de la TB en la comunidad, ampliación de resistencia y una morbimortalidad mayor. Todo esto constituye una barrera para el cumplimiento del Objetivo de Desarrollo Sostenible de controlar la TB para 2030 (3). La duración prolongada del régimen para tratar la TB-RR/MDR, propuesto en las guías de tratamiento del 2011 de la OMS (4), ha impulsado la investigación de regímenes de menos duración con la finalidad de incrementar el éxito del tratamiento y reducir la PEES. Estudios observacionales en Bangladesh (5) y en África (6) aportaron evidencia relevante para que la OMS recomiende acortar el régimen para la TB-RR/MDR a 9-12 meses, lo que en la actualidad se conoce como esquema acortado para TB-RMD. La publicación reciente del estudio STREAM (7) ha determinado mejor las indicaciones de este régimen acortado.

Entre los países con mayor carga de TB resistente en la Región de las Américas, Ecuador ocupa el cuarto lugar con 650 casos anuales estimados de TB-RR/MDR (8). Sin embargo, solo notificó alrededor de 233 casos de TB-RR/MDR en el año 2017, lo que corresponde al 4,16% de la totalidad casos de TB notificados. La alta proporción de condición de egreso como PEES de los casos TB-RR/MDR ha sido una preocupación constante para las autoridades sanitarias en Ecuador. Entre los años 2010 y 2014, como una medida de protección social en salud, el gobierno de turno entregó un incentivo económico condicionado para mejorar la adherencia al tratamiento y reducir la morbimortalidad de las personas con TB-RR/MDR. En un inicio, se observó un efecto favorable con reducción del abandono al tratamiento, pero una vez terminado el proyecto, no se pudo dar sostenibilidad y se tornó, en algunos casos, en un incentivo perverso, ya que los pacientes condicionaban la toma de sus medicamentos a la contribución económica (9, 10). Otra medida adaptada ha sido la implementación, desde noviembre de 2017, del esquema acortado de TB-RR/MDR (OMS) por parte del Ministerio de Salud Pública (MSP) del Ecuador, por lo que se espera reducir la PEES y mejorar el éxito del tratamiento.

Con la finalidad de contribuir con la reducción de la PEES en pacientes TB-RR/MDR en Ecuador, nos planteamos determinar la proporción de PEES en estos pacientes y los factores epidemiológicos y clínicos asociados a esta condición de egreso con respecto a los pacientes egresados como éxito de tratamiento. La determinación de estos factores es importante para la implementación de intervenciones sanitarias dirigidas a mejorar la adherencia al tratamiento en esta población.

## MATERIALES Y MÉTODOS

Ecuador es una república independiente ubicada en el noroeste de América del Sur. Su población es de 17 millones de habitantes y está categorizado como país de ingreso medio-alto por el Banco Mundial. Su tasa anual de incidencia estimada de TB es 43 casos por 100 mil habitantes (2). La atención de la TB se concentra en el sector público y la seguridad social. La provisión del tratamiento de la TB es gratuita y es supervisada en forma directa por el personal de salud. Hasta 2017, la TB-RR/MDR se trataba según las recomendaciones de la Guía de manejo de la TB resistente a medicamentos de la OMS del año 2011 (4). Desde el año 2013, Ecuador ha introducido, en forma progresiva, el diagnóstico de la TB-RR/MDR con la prueba Xpert MTB/Rif^®^ respaldada por la OMS con el financiamiento del Fondo Mundial.

Se trata de un estudio observacional analítico de cohorte retrospectiva de los casos de TB-RR/MDR tratados con el esquema convencional de 18 a 24 meses, diagnosticados entre el 1 de enero de 2014 y el 31 de diciembre de 2015 en Ecuador.

Se consideró la totalidad de casos que fueron notificados a la Estrategia (antes Programa) Nacional de Prevención y Control de la Tuberculosis Ecuador (ENPCTB). Se utilizó la base de datos oficial de la ENPCTB en Excel^®^. Se eliminaron los registros duplicados durante el período de estudio y todos los datos de identificación de la base original de la ENPCTB; en cambio, se usaron códigos únicos de identificación. Se completaron datos no registrados en la base original de la ENPCTB con información obtenida de las fichas de tratamiento de los pacientes, solicitadas a los establecimientos de salud del país.

Para la determinación de la proporción de PEES, se consideró a los pacientes con notificación de condición de egreso: curado, tratamiento completo (ambas suman la categoría éxito de tratamiento), PEES, fallecido, fracaso y no evaluado. Se excluyeron a los pacientes cuya condición final de egreso no fue notificada. Para evaluar los factores asociados a PEES, se consideró a los casos con condición de PEES y éxito de tratamiento (control) y se excluyó a los egresados, como fallecidos, fracasos y no evaluados.

La variable dependiente fue el egreso como PEES: paciente con TB-RR/MDR que interrumpe el tratamiento por lo menos 30 días consecutivos (11).

En cuanto a las variables independientes, se evaluaron aquellas que, de manera programática, se notifican a la ENPCTB. Las fuentes de datos de todas las variables fueron la base de datos electrónica de la ENPCTB y las fichas de tratamiento de cada paciente. Se clasificaron en dos grupos, epidemiológicas y clínicas.

Las características epidemiológicas fueron la edad (años cumplidos), sexo, lugar de residencia (zona sanitaria y provincia), condición de ingreso según el antecedente de tratamiento (nuevo, recaída, PEES recuperado, fracaso), número de tratamientos previos y ser contacto de un caso índice de TB-MDR.

Las características clínicas fueron la localización de la tuberculosis, cultivo de diagnóstico, comorbilidades (virus de inmunodeficiencia humana [VIH], diabetes mellitus [DM], adicción a drogas y alcohol), tipo de resistencia (RMD, RR), presencia de reacciones adversas a los medicamentos, esquema de tratamiento, lugar donde inició el tratamiento (ambulatorio, hospitalización), condición de egreso: éxito (curado más tratamiento completo), PEES, fracaso, muerto y no evaluado; fecha de inicio del tratamiento y fecha de egreso del tratamiento.

### Análisis estadístico

Se determinó la proporción de pacientes TB-RR/MDR según la condición de egreso y el año de inicio de tratamiento. Se compararon las variables independientes entre casos egresados como PEES y los pacientes que egresaron como éxito de tratamiento (curados más tratamiento completo) en un análisis bivariado. La diferencia estadística se evaluó mediante la prueba de chi cuadrado. Se realizó la evaluación cruda y ajustada de los factores de riesgo asociados a la PEES: se consideraron en el modelo de regresión de Cox variables con un valor de asociación bivariado menor a *P* < 0,2 y variables que, según la literatura se asocian a la no adherencia al tratamiento; por ejemplo, sexo masculino, reacciones adversas, adicción a drogas y alcoholismo, entre otras variables evaluadas en este estudio (12-14). Se consideró un valor de *P* < 0,05 como nivel de significación estadística aceptada. Por último, se realizaron curvas de regresión de riesgos proporcionales de Cox, considerando la ocurrencia de PEES como el evento a evaluar en el tiempo. Los datos fueron analizados en Stata 14^®^.

**CUADRO 1.** Condición de egreso de casos de TB-RR/MDR, Ecuador (2014-2015)

Condición de egreso	Año 2014		Año 2015		Total	
	(n = 153)		(n = 117)		(n =270)	
	n	%	n	%	n	%
Curado	70	45,8	47	40,2	117	43,3
Tratamiento completo	12	7,8	7	6,0	19	7,1
PEES	56	36,6	51	43,6	107	396
Muerte	6	3,9	9	7,7	15	5,6
Fracaso	3	2,0	0	0,0	3	1,1
No evaluado	6	3,9	3	2,5	9	3,3

TB-RR/MDR, tuberculosis resistente a rifampicina o resistente a múltiples drogas; PEES, pérdida en el seguimiento.

***Fuente***: elaboración propia con los datos del estudio.

### Consideraciones éticas

El protocolo del estudio fue revisado y aprobado por el Comité de Ética de la Organización Panamericana de la Salud y el Comité de Bioética en Investigación del Hospital de Especialidades “Eugenio Espejo” de Quito, Ecuador, para efectos de resguardar todos los requisitos de investigación en seres humanos. Se resguardó la confidencialidad de los pacientes mediante la eliminación de todos los datos identificativos de la base original con el uso de códigos de identificación. La base de investigación fue accesible solo a los investigadores.

## RESULTADOS

En el año 2014, de 5352 casos de TB notificados en Ecuador, 193 (3,6%) fueron TB-RR/MDR. Para el año 2015, estos mismos valores fueron 5215 y 135 (2,6%), respectivamente. De los 328 casos de TB-RR/MDR notificados en 2014 y 2015, se excluyeron 58 porque no se obtuvo la notificación de la condición final de egreso e ingresaron 270 (82,3%) casos de TB-RR/MDR al análisis. En el cuadro 1 se presenta la condición de egreso de la cohorte histórica según el año de notificación. En promedio, 39,6% de los casos egresaron como PEES y 50,4% tuvieron éxito en el tratamiento.

En el cuadro 2 se comparan las características clínicas y epidemiológicas entre los casos de TB-RR/MDR que egresaron como PEES y los que egresaron como éxito de tratamiento. No hubo diferencia estadística entre ambos grupos en cuanto a la edad de los pacientes, año de notificación, zona de notificación, forma de inicio de tratamiento (ambulatorio u hospitalizado), contacto con casos con TB, tener la comorbilidad con diabetes mellitus, infección por el VIH, localización de la TB, resultado del cultivo de diagnóstico, tipo de resistencia (MDR, RR), uso de inyectables segunda línea o presentar reacción adversa a medicamentos (RAM). Sí se demostró una diferencia estadísticamente significativa entre ambos grupos en su condición de ingreso al tratamiento, sobre todo en de pacientes con historia de haber descontinuado un tratamiento antituberculoso previo y tener adicción al alcohol o drogas. De 20 pacientes ingresados al tratamiento con el antecedente de descontinuar un tratamiento previo (PEES recuperados), el 80% volvieron a tener condición de PEES durante su tratamiento para la TB-RR/MDR. Asimismo, de los cinco pacientes con alcoholismo o adicción a drogas, 100% egresaron del tratamiento como PEES.

El análisis crudo y ajustado de la regresión de Cox para determinar el riesgo de PEES mediante la estimación del cociente de riesgo instantáneo (HR, por sus siglas en inglés) se presenta en el cuadro 3. En el análisis ajustado, las características asociadas estadísticamente a la PEES como condición de egreso fueron: condición de ingreso como PEES recuperado, HR: 2,96 (intervalo de confianza de 95% [IC95%] 1,53-5,73), *P* < 0,001; adicción al alcohol-drogas, HR: 2,82 (IC95% 1,10-7,23), *P* = 0,031 y tener diagnóstico de TB-RR por la prueba Xpert MTB/Rif^®^, HR: 1,53 (IC95% 1,00-2,35), *P* = 0,048.

**FIGURA 1.**
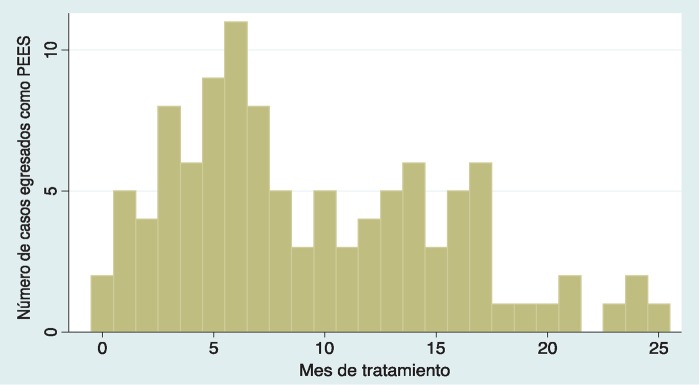
Mes de tratamiento en el que se produjo PEES de los casos de TB-RR/MDR, Ecuador (2014-2015)

En la figura 1 se muestra la frecuencia de casos egresados como PEES respecto al último mes de tratamiento recibido. De 107 casos egresados como PEES, 45 (42%) descontinuaron el tratamiento los primeros 6 meses, 61 (57%) en los 9 meses de tratamiento y 73 (68,2%) el primer año de tratamiento. Cuarenta y tres por ciento de los casos descontinuaron el tratamiento para la TB-RR/MDR después de 9 meses de tratamiento. Hubo dos picos de mayor incidencia de PEES, al sexto mes y al decimocuarto mes.

**FIGURA 2.**
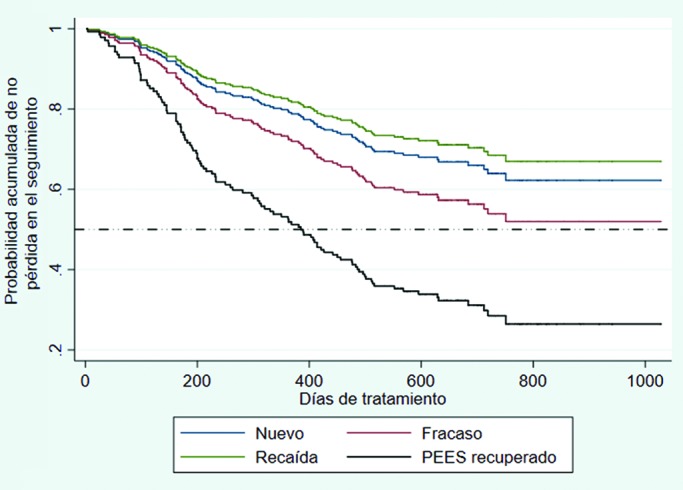
Curva de regresión de riesgo proporcional de Cox de la PEES según condición de ingreso de los casos con TB-RR/MDR, Ecuador (2014-2015)

En la figura 2 se presenta la curva de regresión de riesgos proporcionales de Cox que evalúa la ocurrencia de PEES según la condición de ingreso de los casos de TB-RR/MDR. Se grafica cómo, a diferencia de las otras categorías, ingresar a tratamiento para TB-RR/MDR como PEES recuperado presenta una asociación fuerte con volver a egresar como PEES.

## DISCUSIÓN

El presente estudio incluye el análisis de los datos de 82,3% de los casos de TB-RR/MDR notificados en los años 2014 y 2015 en Ecuador. El 39,6% atribuido a la condición de egreso PEES en esta población está por encima del promedio mundial, con 14% (2) y del promedio de la Región de las Américas, con 26% (8). Según el conocimiento de los autores, este estudio constituye el primer reporte de factores asociados a la PEES en pacientes con TB-RR/MDR en el Ecuador, en un ámbito nacional. Los resultados muestran que tener antecedente de haber descontinuado un esquema previo de tratamiento antituberculoso es el principal factor de riesgo para egresar como PEES. Otros dos factores asociados han sido el antecedente de adicción al alcohol-drogas y haber sido diagnosticado de TB-RR por la prueba Xpert MTB/Rif^®^.

Los pacientes con historia de PEES, antes “abandono al tratamiento”, a un régimen antituberculoso constituyen grupo de riesgo alto para reiterar esta condición de egreso en un subsiguiente régimen de tratamiento (12). También se demostró el aumento de PEES en casos TB tratados antes e inclusive después del tratamiento exitoso anterior (12, 13). Como se mencionó, la falta de compleción del tratamiento casi siempre fue atribuido al paciente como una decisión voluntaria y unilateral. La condición de egreso como “abandono”, que se cambiaba con facilidad a “abandonador”, era un término estigmatizante y sancionador. Por ello, la OMS optó cambiar esta condición de egreso a “pérdida en el seguimiento” para minimizar un lenguaje que culpaba al paciente (1). Esta terminología nueva implica un rol activo y una mayor responsabilidad de los servicios de salud que deben adecuarse a la realidad del paciente, lo que se ha denominado el “enfoque de tratamiento de la TB centrado en el paciente” (15). Las personas que descontinuaron un tratamiento antituberculoso tienen determinantes sociales que condicionan que esta decisión vuelva a repetirse (13, 14). El reto para los sistemas de salud, dentro del nuevo enfoque del Pilar 1 de la Estrategia Fin a la (15) es priorizar acciones y recursos en estos pacientes con riesgo alto de PEES. Al respecto, el reciente Manual de Procedimientos para la Prevención y Control de la Tuberculosis de Ecuador dispone la aplicación de una puntuación de riesgo de PEES al momento del diagnóstico de la TB-RR/MDR. De esta forma, se identificaría en forma temprana a pacientes en riesgo de PEES. Lo que se plantea es la elaboración e implementación de planes de seguimiento del tratamiento individualizados y gerenciados desde el punto de vista social (16).

**CUADRO 2.** Características clínicas y epidemiológicas de casos con TB-RR/MDR egresados como PEES y éxito de tratamiento, Ecuador (2014-2016)

Características	PEES (n = 107)		Éxito de tratamiento (n = 136)		Total (n = 243)		Valor de *P*
	n	%	n	%	n	%	
**Año de estudio**							
2014	56	40,6	82	59,4	138	56,8	0,21
2015	51	48,6	54	51,4	105	43,2	
**Grupos de edad (años)**							
<15	0	0,0	3	100,0	3	1,2	0,41
15-30	37	44,1	47	55,9	84	34,6	
31-45	37	46,8	42	53,2	79	32,5	
46-60	23	47,9	25	52,1	48	19,8	
> 60	10	34,5	19	65,5	29	11,9	
**Sexo**							
Masculino	75	48,7	79	51,3	154	63,4	0,05
Femenino	32	35,9	57	64,1	89	36,6	
**Zona geográfica**							
1^b^	5	38,5	8	61,5	13	5,4	0,72
2^c^	2	66,7	1	33,3	3	1,2	
3^d^	1	25,0	3	75,0	4	1,7	
4^e^	4	40,0	6	60,0	10	4,1	
5^f^	28	56,0	22	44,0	50	20,6	
6^g^	1	50,0	1	50,0	2	0,8	
7^h^	4	36,4	7	64,6	11	4,5	
8^i^	61	41,2	87	58,8	148	60,9	
9^j^	1	50,0	1	50,0	2	0,8	
**Inicio de tratamiento**							
Internado en el hospital	82	45,1	100	54,9	182	74,9	0,58
Ambulatorio	25	41,0	36	59,0	61	25,1	
**Contacto con caso de TB**							
Sí	22	41,5	31	58,5	53	21,8	0,68
No	85	44,7	105	55,2	190	78,2	
**Condición de ingreso**							
Nunca tratado	25	37,9	41	62,1	66	27,2	0,003
PEES recuperado	16	80,0	4	20,0	20	8,2	
Recaída	10	30,3	23	69,7	33	13,6	
Fracaso	56	45,1	68	54,8	124	51,0	
**Diabetes mellitus**							
Sí	33	50,0	33	50,0	66	27,2	0,25
No	74	41,8	103	58,2	177	72,8	
**Infección por VIH**							
Sí	12	60,0	8	40,0	20	8,4	0,13
No	95	42,6	128	57,4	223	91,6	
**Localización de la TB**							
Pulmonar	104	43,7	134	56,3	238	97,9	0,46
Extrapulmonar	3	60,0	2	40,0	5	2,1	
**Cultivo de diagnóstico**							
Positivo	98	91,2	119	87,5	217	89,3	0,61
Negativo	7	38,9	11	61,1	18	7,4	
Sin datos	2	25,0	6	75,0	8	3,3	
**Tipo de resistencia al ingreso**							
TB-RMD (prueba convencional)	54	39,1	84	60,8	138	56,8	0,08
TB-RR (prueba Xpert MTB/Rif®	53	50,5	52	49,5	105	43,2	
**Alcoholismo o adicción a drogas**							
Sí	5	100,0	0	0,0	5	2,1	0,01
No	102	42,9	136	57,1	238	97,9	
**Uso de inyectables de segunda línea^a^**							
Régimen con kanamicina	93	43,8	120	56,3	213	87,6	0,76
Régimen con amikacina	21	55,3	17	44,7	38	15,6	0,13
Régimen con capreomicina	5	33,3	10	66,7	15	6,2	0,39
**Reacción adversa medicamentosa**							
Sí	19	47,5	21	52,5	40	16,5	0,63
No	88	43,4	115	56,7	203	83,5	

^a^ Los pacientes pueden haber recibido más de un inyectable de segunda línea.

^b^ Carchi, Imbabura, Esmeraldas y Sucumbios.

^c^ Napo, Orellana y Pichincha rural.

^d^ Cotopaxi, Chimborazo, Tungurahua y Pastaza.

^e^ Manabí y Santo Domingo de los Tsachilas.

^f^ Los Ríos, Galápagos, Santa Elena, Guayas rural y Bolívar.

^g^ Azuay, Cañar y Morona Santiago.

^h^ Loja, El Oro y Zamora Chinchipe.

^i^ Guayas, Durán y Samborondón.

^j^ Pichincha y Distrito Metropolitano de Quito.

TB, tuberculosis; VIH, virus de la inmunodeficiencia humana; TB-RR/MDR, tuberculosis resistente a rifampicina o resistente a múltiples drogas; PEES, pérdida en el seguimiento; HR, cociente de riesgos instantáneos (por sus siglas en inglés);

***Fuente***: elaboración propia con datos del estudio.

**CUADRO 3.** Factores asociados a PEES en pacientes con TB-RR/MDR, Ecuador (2014-2015)

Factor	Análisis crudo			Análisis ajustado		
	HR	IC 95%	Valor de *P*	HR	IC 95%	Valor de *P*
Sexo masculino	1,47	0,97 – 2,22	0,070	1,24	0,79 – 1,95	0,347
Edad	1,00	0,99 – 1,01	0,843	1,00	0,98 – 1,01	0,966

Fracaso	1,28	0,80 – 2,07	0,305	1,34	0,81 – 2,20	0,254
Recaída	0,78	0,37 – 1,63	0,505	0,85	0,40 – 1,83	0,681
PEES recuperado	2,67	1,42 – 5,04	0,002	2,96	1,53 – 5,73	<0,001
Nuevo	1,00			1,00		
Diabetes mellitus	1,26	0,83 – 1,89	0,278	1,55	0,97 – 2,49	0,068
Infección por VIH	1,80	0,98 – 3,29	0,056	1,52	0,80 – 2,90	0,200
TB-RR (por Xpert MTB/Rif^®^)	1,50	1,03 – 2,20	0,036	1,53	1,00 – 2,35	0,048
Uso de amikacina	1,48	0,91 – 2,42	0,113	1,29	0,78 – 2,14	0,327
Adicción al alcohol o drogas	3,19	1,29 – 7,86	0,012	2,82	1,10 – 7,23	0,031
Reacciones adversas	1,24	0,76 – 2,04	0,389	1,15	0,69 – 1,91	0,600

HR, cociente de riesgos instantáneos (por sus siglas en inglés); IC95%, intervalo de confianza de 95%; TB-RR, tuberculosis resistente a rifampicina; PEES, pérdida en el seguimiento; VIH, virus de la inmunodeficiencia humana.

***Fuente***: elaboración propia con los datos del estudio.

Otro factor asociado a PEES ha sido la adicción al alcohol o drogas. Esto se ha visto en otros reportes que muestran que estas personas tienen menos posibilidades para comenzar o concluir el tratamiento (17-19). En la mayoría de los países con ingresos bajos o medios como Ecuador, el sistema de atención de los pacientes con TB-RR/MDR se sostiene en el primer nivel de atención y la referencia y seguimiento de los casos complejos o pacientes con problemas de salud mental no se efectiviza de manera oportuna. En estas circunstancias, cuando la morbilidad sobrepasa la capacidad de respuesta del primer nivel de atención, el riesgo de la descontinuación del tratamiento a largo plazo es muy alto (20-22).

La comorbilidad con DM también se asoció a una mayor PEES, pero no se obtuvo una significancia estadística, que puede deberse al tamaño de la población evaluada. Algunos estudios demuestran que la DM tiene relación con la presencia de mayor número de RAM a los fármacos de segunda línea (23) y afecta el resultado del tratamiento de TB, con incremento de la mortalidad (24). La DM implica mayor morbilidad, necesidad de mayor número de medicamentos y cuidados por especialistas. Esto, sumado al tiempo y esfuerzo que debe invertir el paciente con DM en el tratamiento de la TB-RR/MDR, aumenta el riesgo para la descontinuación prematura del régimen antituberculoso.

TB-RR/MDR, tuberculosis resistente a rifampicina o resistente a múltiples drogas; PEES, pérdida en el seguimiento.

***Fuente***: elaboración propia con los datos del estudio.

TB-RR/MDR, tuberculosis resistente a rifampicina o resistente a múltiples drogas; PEES, pérdida en el seguimiento.

***Fuente***: elaboración propia con los datos del estudio.

Otro factor asociado a la PEES en nuestra cohorte es haber sido diagnosticado de TB-RR por la prueba Xpert MTB/Rif^®^. El diagnóstico temprano de la TB-RMD, cuando la lesión pulmonar aún no es extensa, y el consecuente inicio de tratamiento apropiado, estaría logrando recuperaciones más rápidas y los pacientes pueden sentirse curados en pocos meses de tratamiento. Esto aumentaría el riesgo de no terminar el tratamiento de 18 a 24 meses, por necesidad de reinsertarse en sus actividades cotidianas. Varias cohortes prospectivas y retrospectivas en Sudáfrica han demostrado que la introducción de la prueba Xpert^®^ no mejoró el resultado del tratamiento y la proporción de PEES continúa siendo alta (25, 26). Este efecto de mayor PEES cuando se introducen pruebas rápidas también se ha visto en Perú, donde luego de la implementación de las pruebas MODS^®^ y Genotype MTBDRplus^®^ se ha producido el incremento de la tasa de la cura y reducción de la mortalidad por TB, pero también un aumento de la PEES como condición de egreso de los pacientes con TB RMD (27).

Un resultado destacado del estudio es haber determinado que casi la mitad de los egresos como PEES sucedieron después de nueve meses de tratamiento. Este hallazgo refuerza la implementación en el país del esquema acortado de nueve meses de la OMS, que reduciría casi la mitad de las PEES de los casos de TB-RR/MDR en el Ecuador. Según el estudio Stream (7), el régimen acortado de nueve meses, cuatro de levofloxacina, kanamicina, clofazimina, protionamida (o etionamida) dosis altas de isoniacida, etambutol y pirazinamida; seguido de 5 meses de levofloxacina, clofazimina, etambutol y pirazinamida; logró una cura de 78,8% en pacientes con TB-RMD con susceptibilidad demostrada a fluoroquinolonas e inyectables de segunda línea, y no demostró ser inferior al esquema convencional de 18 a 24 meses de la OMS. Por ello, consideramos necesario que Ecuador invierta en la implementación de la prueba Genotype MTBDRsl (segunda línea)^®^, que detecta de manera rápida la resistencia a fluroquinolonas e inyectables de segunda línea para una mejor indicación del esquema acortado de la OMS. El tratamiento con el esquema acortado de la OMS no está indicado para las cepas TB preXDR (extensamente resistente, resistencia a fluoroquinolonas e inyectables de segunda línea) o TB-XDR, debido a sus desenlaces clínicos desfavorables y el mayor riesgo de ampliación de la resistencia (28).

Este estudio tuvo limitaciones considerables. Hubo una alta proporción de exclusiones por deficiencia en la notificación de la condición de egreso, lo que demuestra la necesidad de mejorar el sistema de información y seguimiento de estos pacientes. Se realizó un análisis de sensibilidad en el que se consideró que todos los casos egresados como no evaluados fueron PEES o que ninguno de ellos lo fue y no se modificó la significancia estadística de los factores reportados. Nuestro estudio tampoco contempla factores de riesgo atribuidos a la calidad de los servicios de salud ni a la opinión del paciente. Por ejemplo, uno de los factores reconocidos y asociados a la PEES es la percepción de los pacientes de estigma y discriminación por los trabajadores de salud (14, 29) o su desconocimiento sobre la enfermedad, no creer en la curación o no contar con apoyo social. Por la metodología del presente estudio, estos factores cualitativos de los pacientes o del personal de salud no pudieron evaluarse. Otra limitante ha sido la falta de poder estadístico de algunas asociaciones posibles, como la DM, debido al limitado tamaño de muestra, a pesar de considerar más de 80% de los casos de TB-RR/MDR notificados en el país.

En conclusión, la proporción de PEES en pacientes TB-RR/MDR tratados con el esquema de 18-24 meses en Ecuador fue de 39,6%, la cual está por encima del promedio mundial (14%) (2) y en la Región de las Américas (26%) (8). Esta condición de egreso presenta una asociación fuerte con el antecedente de haber descontinuado un tratamiento antituberculoso antes, tener comorbilidades como alcoholismo o drogadicción y haber sido diagnosticado de TB RR por la prueba Xpert MTB/Rif^®^. Aproximadamente, 50% de casos con PEES ocurrieron después de haber recibido nueve meses de tratamiento. Consideramos que los hallazgos de este estudio refuerzan la actual implementación de los esquemas acortados de la OMS en Ecuador y la implementación del tratamiento centrado en el paciente, como los dispone en el Pilar 1 de la Estrategia Fin de la Tuberculosis (15) a la que el país, como miembro de las Naciones Unidas, se ha comprometido cumplir.

## Agradecimientos.

Esta investigación se llevó a cabo mediante la Iniciativa de capacitación estructurada en investigación operativa (SORT IT, por sus siglas en inglés) una alianza mundial dirigida por el Programa Especial de Investigación y Capacitación de Enfermedades Tropicales de la Organización Mundial de la Salud (OMS/TDR) y el Departamento de Enfermedades Transmisibles y Determinantes Ambientales de la Organización Panamericana de la Salud (OPS).

## Financiamiento.

Se obtuvo financiamiento de la oficina subregional Andina de la OPS. Los financiadores no desempeñaron ningún papel en el diseño del estudio, la recopilación y análisis de datos, la decisión de publicar ni la elaboración del estudio.

## Contribución de los autores.

Los autores NTO, AMT y EAA concibieron la idea original. NTO, EAA y JA recopilaron los datos, LL y AMT analizaron los datos. Todos los autores interpretaron los resultados. AMT,NTO y LL escribieron el manuscrito. Todos los autores revisaron y aprobaron la versión final.

## Declaración.

Las opiniones expresadas en este manuscrito son únicamente responsabilidad de los autores y no reflejan necesariamente los de la *Revista Panamericana de Salud Pública* o la Organización Panamericana de la Salud.
